# Facilitators and Barriers to Physical Activity for Patients With Rheumatoid Arthritis and Axial Spondyloarthritis

**DOI:** 10.1111/1756-185X.70109

**Published:** 2025-02-03

**Authors:** Annabel Zhi Yan Lee, Ting Hui Woon, Charmaine Tze May Wang, Yu Heng Kwan, Warren Fong

**Affiliations:** ^1^ Department of Medicine, Yong Loo Lin School of Medicine National University of Singapore Singapore Singapore; ^2^ Department of Rheumatology and Immunology Singapore General Hospital Singapore Singapore; ^3^ Program in Health Services and Systems Research Duke‐NUS Medical School Singapore Singapore; ^4^ Centre for Population Health Research and Implementation SingHealth Regional Health System Singapore Singapore; ^5^ Office of Education Duke‐NUS Medical School Singapore Singapore

## Abstract

**Background:**

Physical activity is important in patients with rheumatoid arthritis (RA) and axial spondyloarthritis (axSpA). Current literature suggests they engage in less physical activity compared to healthy controls. Reasons for this discrepancy has not been well studied in Asian populations. We aimed to understand facilitators and barriers to physical activity in patients with RA and axSpA using the validated Inflammatory arthritis FAcilitators and Barriers (IFAB) questionnaire.

**Methods:**

We conducted a cross‐sectional study from 2020 to 2021 and enrolled patients diagnosed with RA and axSpA. Demographics, disease activity, comorbidities, facilitators, barriers and levels of physical activity were collected. Mean time on recreational activity, facilitators and barriers were listed.

**Results:**

We enrolled 222 patients (109 RA, 113 axSpA). Mean (SD) age was 48.4 (14.8) years and 42.3% were males. Of all patients, 66.7% met the WHO recommendations on physical activity for health and percentage was similar between patients with RA and axSpA. Mean time spent on recreational‐related activities was higher in patients with axSpA. Top three barriers to physical activity included lack of motivation (46.8%), level of symptoms (44.6%) and weather conditions (29.7%). Facilitators included knowledge of the benefits of physical activity for health (68.9%), mood (56.3%), and confidence on how to exercise safely (54.5%).

**Conclusion:**

Overall, the facilitators and barriers to physical activity were similar between patients with RA and axSpA. Physicians could address some of these factors during consultations to promote and encourage more physical activity.

## 1 Introduction

Rheumatoid arthritis (RA) and axial spondyloarthritis (axSpA) are some of the more common inflammatory arthropathies (IA) [1, 2]. These chronic, progressive immune‐mediated rheumatic diseases lead to joint destruction and other systemic manifestations, resulting in functional impairment and a poor quality of life. The joints involved in RA and axSpA are different. RA typically affects the small joints of the hands and feet in a symmetrical distribution, resulting in decreased dexterity, [3] while axSpA typically causes inflammation of the spine, sacroiliac joint, and entheses [4]. Both conditions cause increasing pain and stiffness which can interfere with physical activity [4].

Regular physical activity is a safe and beneficial management option for the physical and mental health in people with IA [5, 6]. Physical activity helps with disease activity and outcomes, and also aids in curtailing the increase risk of comorbidities, such as slowing the atherosclerotic process in cardiovascular disease, increasing bone mineral density and muscle mass, as well as diminishing symptoms of depression, fatigue, and perceived pain [5, 7]. while not aggravating disease activity [8, 9]. In the short term, moderate intensity strength and aerobic exercises help with pain relief, improve physical ability and energy levels, result in better quality sleep and quality of life, [10, 11] while exercise in the long‐term effectively reduces disease activity. In relatively early stages of RA (i.e., ≤ 5 years), it has also been proven to promote clinical remission in these patients [12, 13]. Lower activity levels in those with IA on the other hand has resulted in less than ideal disease outcomes and significant morbidity, with a wider scale impact on healthcare cost and burdens worldwide [14, 15].

Despite the potential benefits, current research reported that patients with IA do not engage in regular physical activity as recommended [6, 16, 17]. These patients often cite fatigue, arthritis‐related pain, and a belief that exercise may worsen arthritis as some of the barriers [6] and the confidence in the ability to exercise, the improvement in energy levels associated with exercise and knowledge of how exercise can benefit health as some of the facilitators [6, 18].

Previous studies looking into the physical activities in patients with IA were conducted in Europe, North Africa, Australia, and Korea [19, 20]. Validated questionnaires such as the International Physical Activity Questionnaire Short form (IPAQ‐S), Global Physical Activity Questionnaire (GPAQ), and Inflammatory arthritis FAcilitators and Barriers (IFAB) questionnaire were used. However, differences in ethnic makeup, geographical context, working and lifestyle habits and disease course and outcomes within the Southeast Asian countries as compared to other Western and Asian counterparts could potentially affect physical activity levels as well as the factors which influence them [21].

Therefore, we aimed to understand the facilitators and barriers of physical activity in axSpA and RA patients in Singapore, and to find out if these facilitators and barriers differed between participants with different physical activity levels, diagnosis, age, and gender. This will aid in the development of targeted interventions to increase physical activity levels in patients with IA, and also further guide and optimize future clinical recommendations in the management of IA patients.

## 2 Materials and Methods

### 2.1 Study Design

A cross‐sectional study was conducted from October 2020 to July 2021, with patients recruited from an outpatient clinic in the Department of Rheumatology and Immunology in Singapore General Hospital, a tertiary hospital in Singapore. Informed consent was obtained from participants and the study was approved by SingHealth Centralized Institutional Review Board (ref no.: 2020/2945).

### 2.2 Participants

Participants were recruited based on the following criteria: (i) patients aged 21 years and above, (ii) clinically diagnosed with axSpA or RA, (iii) patients with axSpA had to fulfill Assessment of Spondyloarthritis International Society (ASAS) Classification Criteria 2009 for axSpA [22] whilst patients with RA had to fulfill ACR/EULAR 2010 Rheumatoid Arthritis Classification Criteria for RA [23]. Patients who were illiterate, cognitively impaired, wheelchair bound, or bedridden were excluded.

### 2.3 Measures Collected

Patient demographics (age, gender, race, years of education, and employment status) and clinical information such as disease activity scores and comorbidities (hypertension, diabetes mellitus, hyperlipidemia, coronary artery disease, chronic kidney disease) were collected. Facilitators, barriers, and levels of physical activity were also collected.

### 2.4 Disease Activity

The disease activity was assessed with scales validated for use in Singapore—Bath Ankylosing Spondylitis Disease Activity Index (BASDAI), [24] Ankylosing Spondylitis Disease Activity Score (ASDAS) [25] and Disease Activity Scale 28‐Erthrocyte Sedimentation Rate (DAS28‐ESR) for Rheumatoid Arthritis [26].

The BASDAI, ASDAS, and DAS28‐ESR score utilized a numeric rating scale. The BASDAI included components such as severity of fatigue, back pain, joint pain, and swelling, localized tenderness and morning stiffness. The ASDAS score was calculated based on the severity of back pain, peripheral pain and swelling and duration of morning stiffness obtained through BASDAI, as well as patient global scores and CRP levels [25]. DAS28‐ESR assessed the number of clinically tender or swollen joints and ESR levels for scoring. For each of these scoring systems, higher scores indicated higher disease activities. The BASDAI sum score ranges from 0 to 10, with scores of 4 or more indicating active disease [27, 28]. Having an ASDAS score of < 1.3 indicates an inactive disease, ≥ 1.3 and < 2.1 indicate a low disease activity, ≥ 2.1 and ≤ 3.5 indicate a high disease activity and a score of > 3.5 indicate very high disease activity [29]. The DAS28‐ESR scores range from 0 to 9.4, where a score of 5.1 or more indicates highly active disease [30, 31].

### 2.5 Facilitators and Barriers of Physical Activity

The barriers and facilitators of physical activities were categorized and documented in the use of the Inflammatory arthritis Facilitators and Barriers to Physical Activity (IFAB) Questionnaire. Each of the items fall into a category of either psychological status, social support, disease, or environmental factors. The IFAB questionnaire was validated specifically for patients with IA. There are a total of 10 items, each scored from 0 to 10, with scores of “0” meaning having no impact on participants' physical activities, and “10” meaning having maximum impact on participants' physical activities. Facilitators of physical activity are scored positively, whilst barriers to physical activities are scored negatively. The combined score ranges from −70 to 70, with a higher score indicating that the item was a greater facilitator and lower score indicating that the items was a greater barrier.

### 2.6 Levels of Physical Activity

The Global Physical Activity Questionnaire (GPAQ) developed by the World Health Organization (WHO) was used to evaluate the level of physical activity as well as sedentary behavior in participants. It contains 16 questions and estimates one's level of physical activity in terms of number of days per week and time spent per day in work, transport, and recreational‐related activities and lastly, perceived time spent in sedentary behavior [32].

WHO defined those with high physical activity levels either having (1) At least 3 days of vigorous intensity activity, meeting minimum 1500 MET‐minutes per week or (2) At least 7 days a combination of walking, moderate‐ or vigorous‐intensity activities, meeting a minimum 3000 MET‐minutes per week.

Participants with moderate levels of physical activity do not meet the criteria of the high activity levels group but have either (1) At least 3 days of vigorous‐intensity activity for a minimum of 20 min per day or (2) At least 5 days of moderate intensity activity or walking for at least 30 min per day or (3) At least 5 days with a combination of walking, moderate‐ or vigorous‐intensity activities, all achieving minimum 600 MET‐minutes per week.

MET refers to metabolic equivalent of task. One MET is defined as the amount of oxygen consumed while at rest, which usually equals to 3.5 mL oxygen uptake/kg/min [33]. While MET‐minutes refers to the number of METs of your activity multiplied by the number of minutes of the exercise performed. It is an indicator of the both the duration and intensity of the exercise [34].

Participants with low levels of physical activity do not meet any of the aforementioned criteria of each category [35].

### 2.7 Statistical Analysis

Data was expressed as mean and standard deviation (SD) for continuous variables to facilitate comparison with other published studies, despite data being mostly non‐parametric. While number and percentages were used for categorical variables. Patients were stratified based on their level of physical activity as defined by the Global Physical Activity Questionnaire (GPAQ) and diagnosis, age and gender. The age range was determined by taking the median age of 50 years from the participants surveyed.

The normality of data was tested using the Shapiro–Wilk test. Continuous variables were compared using Wilcoxon rank sum test or Kruskal–Wallis test as appropriate. Categorical variables were compared using chi‐square test or Fisher exact test as appropriate depending on the sample size. A *p*‐value of < 0.05 was considered statistically significant. All statistical analyses were carried out using STATA SE version 15.0 for Windows (StataCorp, College Station, TX).

## 3 Results

A total of 222 patients were included, of which 109 had RA and 113 had axSpA. Table 1 looks into participant characteristics. Table 2 looks into comparing facilitators and barriers between those with high and moderate physical activity levels and those with low physical activity levels based on the GPAQ. Table 3 looks into comparing facilitators and barriers between RA and axSpA patients.

**TABLE 1:** Participant characteristics.

Characteristics	axSpA (*n* = 113)	RA (*n* = 109)	Total (*N* = 222)	*p*
Current age, mean (S.D.), years	40.1 (12.6)	57 (11.7)	48.4 (14.8)	< 0.01
Male sex, *n* (%)	82 (72.6)	12 (11.0)	94 (42.3)	< 0.01
Race, Chinese, *n* (%)	105 (92.9)	81 (74.3)	186 (83.8)	< 0.01
Body mass index, mean (S.D.), kg/m^2^	25.7 (5.2)	24.9 (5.4)	25.3 (5.3)	0.15
More than 10 years of education, *n* (%)	108 (95.6)	79 (72.5)	187 (84.2)	< 0.01
Employment status				
Employed, *n* (%)	89 (78.8)	65 (59.6)	154 (69.4)	< 0.01
Unemployed, *n* (%)	5 (4.4)	9 (8.3)	14 (6.3)	
Student, *n* (%)	13 (11.5)	1 (0.9)	14 (6.3)	
Housewife, *n* (%)	3 (2.7)	16 (14.7)	19 (8.6)	
Retiree, *n* (%)	3 (2.7)	18 (16.5)	21 (9.5)	
Co‐morbidities				
Hypertension, *n* (%)	16 (14.2)	36 (33.0)	52 (23.4)	< 0.01
Diabetes mellitus, *n* (%)	5 (4.4)	15 (13.8)	20 (9.0)	0.02
Hyperlipidemia, *n* (%)	41 (36.3)	62 (56.9)	103 (46.4)	< 0.01
Coronary artery disease, *n* (%)	3 (2.7)	0 (0)	3 (1.4)	0.25
Chronic kidney disease, *n* (%)	1 (0.9)	1 (0.9)	2 (0.9)	1.00
BASDAI, mean (S.D.)	2.5 (1.9)			
ASDAS^b^, mean (S.D.)	2.0 (0.9)			
DAS28‐ESR, mean (S.D.)		2.7 (1.3)		
Physical activity^a^				
Meets WHO recommendations on physical activity for health, *n* (%)	75 (68.2)	69 (65.1)	144 (66.7)	0.63
Mean time of total physical activity on average per day (S.D.), minutes	88.5 (147.9)	102.2 (137.4)	95.2 (142.7)	0.59
Mean time spent on work‐related physical activities on average per day (S.D.), minutes	42.6 (121.3)	58.5 (124.5)	50.4 (122.9)	0.10
Mean time spent on travel‐related physical activities on average per day (S.D.), minutes	22.8 (43.2)	32.6 (49.7)	27.6 (46.6)	0.08
Mean time spent on recreational‐related physical activities (sport, fitness or recreational activities) on average per day (S.D.), minutes	23.2 (39.2)	11.1 (19.5)	17.3 (31.7)	< 0.01
WHO level of physical activity^a^				
Low level of physical activity, *n* (%)	53 (48.2)	42 (39.6)	95 (44.0)	0.44
Moderate level of physical activity, *n* (%)	35 (31.8)	38 (35.8)	73 (33.8)	
High level of physical activity, *n* (%)	22 (20.0)	26 (24.5)	48 (22.2)	
High level of sedentary activity^c^, *n* (%)	63 (57.3)	31 (29.2)	94 (43.5)	< 0.01

IFAB Item	Total number who rated item as a facilitator, *n* (%)	Total number who rated item as a barrier, *n* (%)	Total number who rated item as having no impact, *n* (%)	*p*
Level of symptoms (pain, fatigue, lack of mobility)	33 (14.9)	99 (44.6)	90 (40.5)	
Weather conditions	16 (7.2)	66 (29.7)	140 (63.1)	
Presence or absence of support from others (friends, family)	74 (33.3)	13 (5.9)	135 (60.8)	
Presence or absence of support and/or advice from healthcare professionals	76 (34.2)	12 (5.4)	134 (60.4)	
A belief that physical activity will make symptoms worse	—	63 (28.4)	159 (71.6)	
Lack of motivation	—	104 (46.8)	118 (53.2)	
Lack of knowledge on which exercises to do and how much	—	46 (20.7)	176 (79.3)	
Knowledge of benefits of physical activity for health	153 (68.9)	—	69 (31.1)	
Knowledge of benefits of physical activity for mood	125 (56.3)	—	97 (43.7)	
Confidence on how to exercise safely	121 (54.5)	—	101 (45.5)	

a Three participants from both RA and SpA groups were excluded from the analysis for physical activity due to erroneous responses.

^b^ Twenty‐seven participants with SpA did not have results for ASDAS as they did not test for CRP levels.

^c^ Defined as more than 8 h of sedentary activity.

**TABLE 2:** Facilitators and Barriers questionnaire (IFAB), grouped by levels of physical activity based on Global Physical Activity Questionnaire (GPAQ) (*n* = 216).^a^

Item	Number of participants who rated item as a facilitator, *n* (%)		Number of participants who rated item as a barrier, *n* (%)		Number of participants who rated item as having no impact, *n* (%)			Score of items, mean (S.D.)		
	High and moderate levels of physical activity (*n* = 121)	Low levels of physical activity (*n* = 95)	High and moderate levels of physical activity (*n* = 121)	Low levels of physical activity (*n* = 95)	High and moderate levels of physical activity (*n* = 121)	Low levels of physical activity (*n* = 95)	*p*	High and moderate levels of physical activity	Low levels of physical activity	*p*
Facilitators or barriers										
Level of symptoms (pain, fatigue, lack of mobility)	19 (15.7)	13 (13.7)	54 (44.6)	44 (46.3)	48 (39.7)	38 (40)	0.91	−1.7 (4.3)	−2.2 (4.3)	0.61
Weather conditions	7 (5.8)	8 (8.4)	37 (30.6)	28 (29.5)	77 (63.6)	59 (62.1)	0.75	−1.3 (3.5)	−1.5 (3.6)	0.92
Presence or absence of support from others (friends, family)	41 (33.9)	32 (33.7)	7 (5.8)	6 (6.3)	73 (60.3)	57 (60)	0.99	2.0 (3.7)	1.9 (3.6)	0.78
Presence or absence of support and/or advice from healthcare professionals	41 (33.9)	31 (32.6)	8 (6.6)	4 (4.2)	72 (59.5)	60 (63.2)	0.71	2.0 (3.8)	1.8 (3.3)	0.77
Barriers										
A belief that physical activity will make symptoms worse			37 (30.6)	24 (25.3)	84 (69.4)	71 (74.7)	0.39	−1.7 (2.8)	−1.5 (2.9)	0.48
Lack of motivation			54 (44.6)	49 (51.6)	67 (55.4)	46 (48.4)	0.31	−2.5 (3.1)	−3.2 (3.6)	0.12
Lack of knowledge on which exercises to do and how much			24 (19.8)	21 (22.1)	97 (80.2)	74 (77.9)	0.68	−0.9 (2.1)	−1.2 (2.4)	0.33
Facilitators										
Knowledge of benefits of physical activity for health	87 (71.9)	62 (65.3)			34 (28.1)	33 (34.7)	0.30	4.8 (3.4)	4.4 (3.6)	0.50
Knowledge of benefits of physical activity for mood	66 (54.5)	54 (56.8)			55 (45.4)	41 (43.2)	0.74	3.5 (3.5)	3.8 (3.7)	0.45
Confidence on how to exercise safely	69 (57.0)	48 (50.5)			52 (43.0)	47 (49.5)	0.34	3.7 (3.6)	3.6 (3.8)	0.83
Global IFAB score								7.9 (15.1)	6.0 (15.8)	0.41

^a^ Six participants were excluded from this analysis due to erroneous responses for GPAQ.

**TABLE 3:** Inflammatory arthritis Facilitators and Barriers questionnaire (IFAB), grouped by diagnosis.

Category	Item	Number of participants who rated item as a facilitator, *n* (%)		Number of participants who rated item as a barrier, *n* (%)		Number of participants who rated item as having no impact, *n* (%)			Score of items, mean (S.D.)		
		axSpA (*n* = 113)	RA (*n* = 109)	axSpA (*n* = 113)	RA (*n* = 109)	axSpA (*n* = 113)	RA (*n* = 109)	*p*	axSpA (*n* = 113)	RA (*n* = 109)	*p*
Facilitators or barriers	Level of symptoms (pain, fatigue, lack of mobility)	20 (17.7)	13 (11.9)	49 (43.4)	50 (45.9)	44 (38.9)	46 (42.2)	0.48	−1.5 (4.0)	−2.3 (4.5)	0.16
	Weather conditions	6 (5.3)	10 (9.2)	29 (25.7)	37 (33.9)	78 (69)	62 (56.9)	0.16	−1.1 (2.8)	−1.7 (4.1)	0.21
	Presence or absence of support from others (friends, family)	29 (25.7)	45 (41.3)	7 (6.2)	6 (5.5)	77 (68.1)	58 (53.2)	0.05	1.4 (3.3)	2.4 (3.9)	0.03
	Presence or absence of support and/or advice from healthcare professionals	32 (28.3)	44 (40.4)	6 (5.3)	6 (5.5)	75 (66.4)	59 (54.1)	0.16	1.6 (3.0)	2.3 (4.0)	0.07
Barriers	A belief that physical activity will make symptoms worse			30 (26.5)	33 (30.3)	83 (73.5)	76 (69.7)	0.54	−1.5 (2.8)	−1.7 (2.9)	0.54
	Lack of motivation			56 (49.6)	48 (44.0)	57 (50.4)	61 (56)	0.41	−2.8 (3.3)	−2.7 (3.3)	0.63
	Lack of knowledge on which exercises to do and how much			23 (20.4)	23 (21.1)	90 (79.6)	86 (78.9)	0.89	−0.9 (2.2)	−1.1 (2.3)	0.86
Facilitators	Knowledge of benefits of physical activity for health	78 (69.0)	75 (68.8)			35 (31.0)	34 (31.2)	0.97	4.4 (3.5)	4.7 (3.5)	0.65
	Knowledge of benefits of physical activity for mood	61 (54.0)	64 (58.7)			52 (46.0)	45 (41.3)	0.48	3.4 (3.6)	4 (3.6)	0.30
	Confidence on how to exercise safely	60 (53.1)	61 (56.0)			53 (46.9)	48 (44.0)	0.67	3.5 (3.7)	3.8 (3.7)	0.42
	Global IFAB score								6.6 (14.4)	7.8 (16.2)	0.34

The mean (SD) age of all participants surveyed was 48.4 (14.8) years, with patients with axSpA being younger than the RA group [40.1 (12.6) years vs. 57.0 (11.7) years, respectively, *p* < 0.01]. There were more males (*n* = 82, 72.6% vs. *n* = 12, 11.0%, respectively, *p* < 0.01) and Chinese (*n* = 105, 92.9% vs. *n* = 81, 74.3%) in patients with axSpA compared to RA. More patients with RA had hypertension (*n* = 36, 33.0% vs. *n* = 16, 14.2%, *p* < 0.01), diabetes mellitus (*n* = 15, 13.8% vs. *n* = 5, 4.4%, *p* = 0.02) and hyperlipidemia (*n* = 62, 56.9% vs. *n* = 41, 36.3%, *p* < 0.01).

### 3.1 Levels of Physical Activity

About two‐thirds (*n* = 144, 66.7%) of all patients met the WHO recommendations on physical activity for health. Mean (SD) time of total physical activity on average per day was higher in patients with RA compared to axSpA [102.2 min (137.4) vs. 88.5 min (147.9) respectively, *p* = 0.59], but the difference was not statistically significant. Mean time spent on work‐related physical activities and travel‐related activities was similar between both disease groups. However, patients with axSpA spent significantly more time on recreational‐related physical activities per day compared to patients with RA [23.2 min (39.2) vs. 11.1 min (19.5), *p* < 0.01]. Majority of patients (*n* = 168, 77.8%) participated in low to moderate levels of physical activity. Sixty‐three (*n* = 63, 57.3%) patients with axSpA had high levels of sedentary activity, which was significantly greater than that observed in patients with RA [*n* = 31, 29.2%, *p* < 0.01].

### 3.2 Barriers to Physical Activity

The top three barriers to participation in physical activity included a lack of motivation (*n* = 104, 46.8%), level of symptoms which indicates the severity of symptoms (*n* = 99, 44.6%) and weather conditions (*n* = 66, 29.7%) (Table 1). These were also the top three barriers identified among participants with different levels of physical activity (Table 2). When stratified by diagnosis, this was similar in patients with RA, while having a belief that physical activity will worsen symptoms instead of weather conditions was the third most common barrier in patients with axSpA (*n* = 30, 26.5%) (Table 3). When grouped by age and gender, the top three barriers were similar for those age > 50 and females, while the belief that physical activity will worsen symptoms instead of weather conditions, like patients with axSpA, was also the third barrier in patients age ≤ 50 (*n* = 37, 32.5%) and males (*n* = 28, 29.8%) (Tables S1 and S2).

### 3.3 Facilitators of Physical Activity

Top three facilitators of physical activity included knowledge of the benefits of physical activity for health (*n* = 153, 68.9%) and mood (*n* = 125, 56.3%) as well as confidence on how to exercise safely (*n* = 121, 54.5%) (Table 1). These were also the top three facilitators identified among participants with different levels of physical activity (Table 2), diagnosis (Table 3), age (Table S1) and gender (Table S2). It was also noted that more female participants rated the presence or absence of support from family and friends [*n* = 56, 43.8% vs. *n* = 18, 19.1%, *p* < 0.01] as well as healthcare professionals [*n* = 55, 43.0% vs. *n* = 21, 22.3%, *p* < 0.01] as being facilitators of physical activity. However, more male participants rated these as having no impact [*n* = 70, 74.5% vs. *n* = 65, 50.8%, *p* < 0.01; *n* = 65, 69.1% vs. *n* = 69, 53.9%, *p* < 0.01] (Table S2).

## 4 Discussion

In this study, we identified the facilitators and barriers to physical activity in patients with RA and axSpA. Patients with RA were older, predominantly female, and had more comorbidities than patients with axSpA. While patients with RA spent more time on average per day on work and travel‐related activities, patients with axSpA spent more time on recreational‐related physical activities.

Overall, the lack of motivation, level of symptoms and weather conditions were the top three barriers which discouraged participants from being involved in physical activity. Based on similar studies, level of symptoms and lack of motivation were also significant barriers to exercise, while a study in Australia noted that a proportion of respondents cited environmental and situational barriers such as weather conditions, in addition to the aforementioned factors as a significant barrier [18, 20, 36, 37, 38].

Knowledge of the benefits of physical activity for health and mood, and the confidence on how to exercise safely were the top three facilitators for patients to engage in physical activity, regardless of different levels of physical activity and diagnosis. Improvement in symptoms and health were also commonly reported as facilitators to physical activity as well in other studies [38]. However, as compared to other studies, social motivation and being aware of the benefits of physical activity was less of a facilitator for patients with RA in Western countries like London [39].

The public's perception and attitude towards health and healthcare and thus facilitators and barriers are shaped by the cultural, societal, and geographical context of Singapore being a smaller, population dense, tropical and multi‐ethnic country. In Singapore, value is placed on familial and community support, yet at the same time fast‐paced and high stress environments affect time with loved ones and energy levels to engage in physical activity. However, with the government's recent promotion of Healthier SG [40] and The Singapore Green Plan 2030, [41] trends are now observed on a macro level where there is now greater access to green spaces and exercise corners. Preliminary population health surveys indicate that with the implementation of these policies, overall physical activity in the local population has increased [42]. However, the longer term impact of these initiatives have yet to be fully understood.

The presence of support from friends and family, as well as reinforcement from healthcare professionals were more significant facilitators of physical activity in female patients in our population. Studies have investigated how support from healthcare professionals have facilitated greater physical activity engagement in females with other medical conditions [43]. One probability is that females, especially Asians, place greater importance in having supportive communities and peer motivation [44]. It has been highlighted in previous studies that cultural differences results in different emotional processing styles, with interdependence being a driving force for many Asians [17, 45]. Social reinforcement from healthcare professionals, involvement of caregivers in patient care and group exercise programmes which are tailored to patients' abilities may be useful to facilitate uptake. By providing an environment to promote exercising safely, this may help to increase subsequent long‐term adherence to physical activity among the female population.

The lack of significance in levels of physical activity between the RA and axSpA group based on the Global IFAB score could be due to the subjectivity of understanding and thus under or overreporting of actual physical activity levels. Additionally, the score has mainly be tried and tested in European countries and are subjected to interpretation based on the geographical and cultural environment [46] The study could benefit from a larger sample size with further refinement in the validity of Global IFAB in the Southeast Asian context.

There are other limitations to our study. Firstly, given the cross‐sectional nature of the study, we cannot draw causal relationships between the facilitators and barriers with the physical activity levels. Secondly, although we used GPAQ, which is a validated tool, as a measure of physical activity, it is open to recall bias as compared to other objective markers such as step trackers and metabolic equivalent of task scores (MET‐min/week). A prospective longitudinal cohort study will be helpful in determining the causal association between physical activity levels and that of the respective facilitators and barriers, while correlating and quantifying time spent on physical activity in patients with axSpA and RA. Thirdly, there was no data collected on the context in which our participants received formal education on the condition from and thus perceptions on the benefits of physical exercise on clinical outcomes may differ.

Nevertheless, a better understanding of the facilitators and barriers to physical activity for patients with axSpA and RA is essential for building more targeted and personalized systems to incorporating physical activity into patients' daily lives. The early introduction of tailored and gradated exercise prescriptions including specific type and dosages of exercise [6] guided by but not limited to the recommendation of guidelines such as EULAR or American College of Sport Medicine‐American Heart Association (ACSM‐AHA) can be pivotal in initiating effective exercise therapy from the point of diagnosis [47, 48].

Improving patient education to correct misconceptions and highlighting the advantages of physical activity for health, mood, symptom management, and structured programmes can significantly enhance IA management, particularly for female patients. By integrating various strategies, such as wearable devices, visual exercise demonstrations to address safety concerns with exercise in IA, and group‐based programmes, this approach could ultimately boost the uptake and sustained participation in physical activity among the IA population [49, 50].

## Author Contributions

A.Z.Y.L., T.H.W., C.T.M.W., Y.H.K. and W.F. conceived and designed this study. T.H.W., C.T.M.W. and W.F. acquired the data for this study. A.Z.Y.L., T.H.W., Y.H.K. and W.F. performed the analyses and prepared the first draft of the manuscript. A.Z.Y.L., T.H.W., C.T.M.W., Y.H.K. and W.F. interpreted the results. All authors read, revised critically, and approved the final manuscript.

## Conflicts of Interest

The authors declare no conflicts of interest.

## Supporting information


Table S1.

## Data Availability

Research data are not shared.
