# Supplementary material for: Facilitators and Barriers to Physical Activity for Patients With Rheumatoid Arthritis and Axial Spondyloarthritis
Source: Int J Rheum Dis. 2025 Feb 3;28(2):e70109. doi: 10.1111/1756-185X.70109 (PMC11788881; doi:10.1111/1756-185X.70109)
Supplement: Supplementary file 1 — Table S1. [file APL-28-e70109-s001.docx]

**Supplementary Table 1**. Inflammatory arthritis Facilitators And Barriers questionnaire (IFAB), grouped by age

|  |  | Number of participants who rated item as a facilitator, n (%) | | Number of participants who rated item as a barrier, n (%) | | Number of participants who rated item as having no impact, n (%) | |  | Score of items, mean (S.D.) | | |
| --- | --- | --- | --- | --- | --- | --- | --- | --- | --- | --- | --- |
|  |  | Age ≤50 (n=114) | Age >50 (n=108) | Age ≤50 (n=114) | Age >50 (n=108) | Age ≤50 (n=114) | Age >50 (n=108) | p-value | Age ≤50 (n=114) | Age >50 (n=108) | p-value |
| Facilitators or barriers | Level of symptoms (pain, fatigue, lack of mobility) | 18 (15.8) | 15 (13.9) | 57 (50) | 42 (38.9) | 39 (34.2) | 51 (47.2) | 0.14 | -2.1 (4.2) | -1.7 (4.4) | 0.39 |
|  | Weather conditions | 8 (7) | 8 (7.4) | 34 (29.8) | 32 (29.6) | 72 (63.2) | 68 (63) | 0.99 | -1.2 (3.1) | -1.5 (3.8) | 0.71 |
|  | Presence or absence of support from others (friends, family) | 31 (27.2) | 43 (39.8) | 9 (7.9) | 4 (3.7) | 74 (64.9) | 61 (56.5) | 0.08 | 1.5 (3.6) | 2.4 (3.6) | 0.05 |
|  | Presence or absence of support and/or advice from healthcare professionals | 29 (25.4) | 47 (43.5) | 7 (6.1) | 5 (4.6) | 78 (68.4) | 56 (51.9) | 0.02 | 1.3 (3.1) | 2.6 (3.9) | <0.01 |
| Barriers | A belief that physical activity will make symptoms worse |  |  | 37 (32.5) | 26 (24.1) | 77 (67.5) | 82 (75.9) | 0.17 | -1.7 (2.9) | -1.5 (2.9) | 0.27 |
|  | Lack of motivation |  |  | 59 (51.8) | 45 (41.7) | 55 (48.2) | 63 (58.3) | 0.13 | -2.9 (3.2) | -2.6 (3.4) | 0.34 |
|  | Lack of knowledge on which exercises to do and how much |  |  | 24 (21.1) | 22 (20.4) | 90 (78.9) | 86 (79.6) | 0.9 | -0.9 (2.1) | -1.1 (2.4) | 0.72 |
| Facilitators | Knowledge of benefits of physical activity for health | 80 (70.2) | 73 (67.6) |  |  | 34 (29.8) | 35 (32.4) | 0.68 | 4.6 (3.5) | 4.5 (3.5) | 0.92 |
|  | Knowledge of benefits of physical activity for mood | 65 (57) | 60 (55.6) |  |  | 49 (43) | 48 (44.4) | 0.83 | 3.6 (3.6) | 3.7 (3.6) | 0.95 |
|  | Confidence on how to exercise safely | 61 (53.5) | 60 (55.6) |  |  | 53 (46.5) | 48 (44.4) | 0.76 | 3.5 (3.6) | 3.8 (3.8) | 0.39 |
|  | Global IFAB score |  |  |  |  |  |  |  | 5.7 (14.7) | 8.8 (15.8) | 0.11 |

**Supplementary Table 2.** Inflammatory arthritis Facilitators And Barriers questionnaire (IFAB), grouped by gender

|  | Item | Number of participants who rated item as a facilitator, n (%) | | Number of participants who rated item as a barrier, n (%) | | Number of participants who rated item as having no impact, n (%) | |  | Score of items, mean (S.D.) | | |
| --- | --- | --- | --- | --- | --- | --- | --- | --- | --- | --- | --- |
|  |  | Male (n=94) | Female (n=128) | Male (n=94) | Female (n=128) | Male (n=94) | Female (n=128) | p-value | Male (n=94) | Female (n=128) | p-value |
| Facilitators or barriers | Level of symptoms (pain, fatigue, lack of mobility) | 17 (18.1) | 16 (12.5) | 40 (42.5) | 59 (46.1) | 37 (39.4) | 53 (41.4) | 0.51 | -1.5 (3.8) | -2.1 (4.6) | 0.19 |
|  | Weather conditions | 6 (6.4) | 10 (7.8) | 24 (25.5) | 42 (32.8) | 64 (68.1) | 76 (59.4) | 0.41 | -0.9 (2.6) | -1.7 (4.0) | 0.19 |
|  | Presence or absence of support from others (friends, family) | 18 (19.1) | 56 (43.8) | 6 (6.4) | 7 (5.5) | 70 (74.5) | 65 (50.8) | <0.01 | 0.9 (2.9) | 2.7 (3.9) | <0.01 |
|  | Presence or absence of support and/or advice from healthcare professionals | 21 (22.3) | 55 (43.0) | 8 (8.5) | 4 (3.1) | 65 (69.1) | 69 (53.9) | <0.01 | 1.0 (3.1) | 2.7 (3.7) | <0.01 |
| Barriers | A belief that physical activity will make symptoms worse |  |  | 28 (29.8) | 35 (27.3) | 66 (70.2) | 93 (72.7) | 0.69 | -1.7 (3.0) | -1.5 (2.8) | 0.67 |
|  | Lack of motivation |  |  | 43 (45.7) | 61 (47.7) | 51 (54.3) | 67 (52.3) | 0.78 | -2.5 (3.1) | -2.9 (3.4) | 0.56 |
|  | Lack of knowledge on which exercises to do and how much |  |  | 17 (18.1) | 29 (22.7) | 77 (81.9) | 99 (77.3) | 0.41 | -0.9 (2.1) | -1.1 (2.4) | 0.59 |
| Facilitators | Knowledge of benefits of physical activity for health | 60 (63.8) | 93 (72.7) |  |  | 34 (36.2) | 35 (27.3) | 0.16 | 4.0 (3.6) | 5.0 (3.4) | 0.07 |
|  | Knowledge of benefits of physical activity for mood | 51 (54.3) | 74 (57.8) |  |  | 43 (45.7) | 54 (42.2) | 0.60 | 3.2 (3.4) | 4.0 (3.7) | 0.12 |
|  | Confidence on how to exercise safely | 51 (54.3) | 70 (54.7) |  |  | 43 (45.7) | 58 (45.3) | 0.95 | 3.4 (3.6) | 3.8 (3.7) | 0.48 |
|  | Global IFAB score |  |  |  |  |  |  |  | 5.0 (13.5) | 8.8 (16.3) | 0.05 |
